# Intrathoracic versus Cervical ANastomosis after minimally invasive esophagectomy for esophageal cancer: study protocol of the ICAN randomized controlled trial

**DOI:** 10.1186/s13063-016-1636-2

**Published:** 2016-10-18

**Authors:** Frans van Workum, Stefan A. W. Bouwense, Misha D. P. Luyer, Grard A. P. Nieuwenhuijzen, Donald L. van der Peet, Freek Daams, Ewout A. Kouwenhoven, Marc J van Det, Frits J. H. van den Wildenberg, Fatih Polat, Suzanne S. Gisbertz, Mark I. van Berge Henegouwen, Joos Heisterkamp, Barbara S. Langenhoff, Ingrid S. Martijnse, Janneke P. Grutters, Bastiaan R. Klarenbeek, Maroeska M. Rovers, Camiel Rosman

**Affiliations:** 1 Department of Surgery, Radboudumc, PO Box 9101, 6500 HB Nijmegen, The Netherlands; 2 Department of Surgery, Catharina Hospital, PO Box 1350, 5602 ZA Eindhoven, The Netherlands; 3 Department of Surgery, VU University Medical Centre, PO Box 7057, 1007 MB Amsterdam, The Netherlands; 4 Department of Surgery, Ziekenhuisgroep Twente, PO Box 7600, 7600 SZ Almelo, The Netherlands; 5 Department of Surgery, Canisius-Wilhelmina Hospital, PO Box 9015, 6500 GS Nijmegen, The Netherlands; 6 Department of Surgery, Academic Medical Centre, PO Box 22660, 1100 DD Amsterdam, The Netherlands; 7 Department of Surgery, Elisabeth-Tweesteden Hospital, PO Box 90151, 5000 LC Tilburg, The Netherlands; 8 Department of Health Evidence, Radboudumc, PO Box 9101, 6500 HB Nijmegen, The Netherlands

**Keywords:** Minimally invasive esophagectomy, Esophageal carcinoma, Intrathoracic anastomosis, Cervical anastomosis, Anastomotic leakage, Dysphagia, Quality of life

## Abstract

**Background:**

Currently, a cervical esophagogastric anastomosis (CEA) is often performed after minimally invasive esophagectomy (MIE). However, the CEA is associated with a considerable incidence of anastomotic leakage requiring reintervention or reoperation and moderate functional results. An intrathoracic esophagogastric anastomosis (IEA) might reduce the incidence of anastomotic leakage, improve functional results and reduce costs. The objective of the ICAN trial is to compare anastomotic leakage and postoperative morbidity, mortality, quality of life and cost-effectiveness between CEA and IEA after MIE.

**Methods/design:**

The ICAN trial is an open randomized controlled multicentre superiority trial, comparing CEA (control group) with IEA (intervention group) after MIE. All patients with esophageal cancer planning to undergo curative MIE are considered for inclusion. A total of 200 patients will be included in the study and randomized between the groups in a 1:1 ratio. The primary outcome is anastomotic leakage requiring reintervention or reoperation, and secondary outcomes are (amongst others) other postoperative complications, new onset of organ failure, length of stay, mortality, benign strictures requiring dilatation, quality of life and cost-effectiveness.

**Discussion:**

We hypothesize that an IEA after MIE is associated with a lower incidence of anastomotic leakage requiring reintervention or reoperation than a CEA. The trial is also designed to give answers to additional research questions regarding a possible difference in functional outcome, quality of life and cost-effectiveness.

**Trial registration:**

Netherlands Trial Register: NTR4333. Registered on 23 December 2013.

## Background

The annual incidence of esophageal carcinoma is increasing [1]. Anastomotic leakage is an important early postoperative complication that often results in reoperation, delayed (ICU) discharge, psychological distress and associated costs. Leak rates cited in the recent literature range from 0–30 % [2, 3], and anastomotic leakage is the main cause of postoperative mortality. It is estimated to be responsible for up to 40 % of deaths following esophagectomy and is associated with a prolonged ICU treatment and hospital stay [3, 4]. Late complications, such as anastomotic stricture, are also responsible for significant morbidity. Stricture rates following esophageal anastomosis range from 5–40 % [5].

In current practice, both intrathoracic esophagogastric anastomosis (IEA) and cervical esophagogastric anastomosis (CEA) are used worldwide to restore gastrointestinal continuity after esophagectomy with gastric tube reconstruction. Four randomized controlled trials including a total of 267 patients have been performed that compared the results of CEA with IEA after open esophagectomy [2]. The mean incidence of anastomotic leakage was 26 % after CEA and 4 % after IEA. No significant differences between the groups were found for mortality, pulmonary complications or hospital stay.

However, all trials performed so far have serious methodological flaws. All trials are single centre studies including a limited number of 32 to 92 patients, of whom a substantial number was either excluded from the analysis for unclear reasons or not reported. Furthermore, allocation concealment was not always reported and intention-to-treat analyses not performed. All studies were performed before neoadjuvant chemoradiotherapy was introduced, and none of the studies included patients undergoing minimally invasive esophagectomy (MIE). Finally, data on quality of life, functional results and cost-effectiveness have not been studied.

Therefore, the results of the previous trials are difficult to interpret and may not be applicable to current surgical practice. Since high-quality evidence is lacking, there are no (inter)nationally accepted evidence-based guidelines for the preferred location of the esophagogastric anastomosis.

## Methods/design

### Study aim

The aim of the ICAN trial is to compare anastomotic leakage and postoperative morbidity, mortality, quality of life and cost-effectiveness between CEA (control group) and IEA (intervention group) after MIE.

### Design and setting

The ICAN trial is designed as an open randomized, multicentre superiority trial. Patients are randomly allocated to undergo MIE with CEA or IEA (Table 1). The study will be conducted in Dutch hospitals with surgeons who are competent in performing both techniques.

**Table 1:** Schedule of enrolment, interventions and assessments

		Study period					
	Enrolment and allocation	Post-allocation					Close-out
Timepoint (months)		Surgery	Follow-up after surgery				
	0	1	2.5	4	7	13	25
Enrolment:							
Eligibility screen	X						
Informed consent	X						
Allocation	X						
Interventions:							
MIE with cervical anastomosis		X					
MIE with intrathoracic anastomosis		X					
Assessments:							
Baseline patient characteristics	X						
Baseline tumour characteristics	X						
QOL questionnaires	X		X	X	X	X	X
CE questionnaires	X			X	X	X	X
Postoperative outcome data		X					
Pathology data		X					
Functional recovery data		X	X	X	X	X	X
Oncologic follow-up data			X	X	X	X	X

*QOL* quality of life, *CE* cost-effectiveness

### Primary endpoint

The primary endpoint is anastomotic leakage for which endoscopic, radiologic or surgical reintervention is needed. This corresponds to the Esophagectomy Complications Consensus Group (ECCG) definition of anastomotic leakage type II and type III [6]. Anastomotic leakage is defined according to the ECCG [6] as a full thickness defect of the anastomosis. This can be identified by (1) computed tomography (CT) scan with intravenous and oral contrast (’swallow CT scan’), (2) endoscopy, (3) drainage of ingested materials into the chest tube (intervention group) or ingested materials or saliva into the cervical wound (control group) or (4) established anastomotic leakage during reintervention, reoperation or autopsy. Anastomotic leakage is assessed during treatment by the treating surgeon and a member of the ICAN study team. In addition, a third ICAN study team member will assess anastomotic leakage using blinded medical reports. Leakages of the gastric tube are scored separately and will be reported separately. If the location of a leak is uncertain (anastomosis or gastric tube), but it has been shown that a leak is present, this is scored as anastomotic leakage.

### Secondary endpoints

Secondary endpoints are incidence of postoperative complications (i.e. pneumonia, pneumothorax, pleural empyema, mediastinal abscess, cardiac complications), severity of complications according to the modified Clavien-Dindo classification [7] and new onset organ failure according to the Sepsis-related Organ Failure Assessment (SOFA) score [8]. General quality of life is scored by EuroQol 5D, whereas cancer-specific quality of life is assessed with the European Organization for Research and Treatment of Cancer Quality of Life Questionnaire (EORTC-QLQ C30). To assess esophageal cancer-specific quality of life, the EORTC-QLQ OG25 module is used. From the questionnaires, the domains of general quality of life and eating-related quality of life are the most important outcomes, although all domains of the questionnaires will be assessed.

Functional outcome parameters, including dysphagia, regurgitation, weight loss, return to preoperative weight and the incidence of benign strictures requiring dilatation are also scored. Other secondary endpoints are hospital mortality, mortality after 30 and 90 days, length of stay in the ICU and in the hospital, ICU and hospital readmission rate, the incidence of recurrent laryngeal nerve trauma and cost-effectiveness.

### Population

All patients with resectable esophageal carcinoma are screened for eligibility by their esophageal surgeon.

### Inclusion criteria

Inclusion criteria are age ≥18 years and histologically proven primary esophageal adenocarcinoma or squamous cell carcinoma. Patients are only included if the tumour is considered resectable (cT1b-4a, N0-3, M0) and the bulk of the tumour is located in the distal or mid esophagus (distal to the level of the carina) or at the level of the cardia-esophageal junction (up to Siewert II [9]).

### Exclusion criteria

Exclusion criteria are previous major gastric or major thoracic surgery rendering MIE unfeasible, prognosis determining malignancy other than esophageal cancer, inability to undergo curative resection and/or follow-up and inability to provide oral or written informed consent.

### Time of inclusion and randomization

In the Netherlands, most patients with resectable tumours are scheduled to undergo neoadjuvant chemoradiotherapy, which is completed approximately 8 weeks before surgery. Patients will be given information about the trial and will be randomized after neoadjuvant chemoradiotherapy or in the weeks before surgery if neoadjuvant chemoradiotherapy is not indicated.

### Randomization

Patients will be randomized to the intervention group or the control group with a 1:1 ratio, using a computerized randomization tool (Castor [10]). Patients are stratified according to hospital in order to ensure that other local treatment factors (i.e. postoperative care) are unlikely to interfere with study outcome. Permuted-block randomization with varying block size is used. Allocation and block size are concealed to all investigators. Because of the nature of this trial involving different surgical incisions, blinding treating surgeons and trial participants is unfeasible: A patient will feel whether a cervical incision has been made, and the surgeon knows which operation has been performed. However, a study team member will assess blinded medical reports regarding anastomotic leakage in order to obtain blinded assessment of the primary outcome parameter.

Patients can withdraw from the study at any time. The operating surgeon can decide not to perform a resection during surgery if the tumour is found to be irresectable because of ingrowth into adjacent organs, if perioperative metastases to other organs are found or if resection is unfeasible because of other perioperative events or findings. Modification of the intervention during surgery is justified if the operating surgeon decides he is unable to perform the allocated anastomosis. In this case, follow-up continues according to the trial protocol, and an intention-to-treat analysis will be performed.

### Operative technique

All patients will be scheduled to undergo MIE with IEA or CEA. For both treatment arms, an identical two-field lymphadenectomy is performed, and a gastric tube reconstruction with the gastric tube in the posterior mediastinum is used. An omental wrap is performed after IEA and CEA, since there is evidence that this reduces the incidence of clinically relevant anastomotic leakage [11]. Other surgical factors are left to the discretion of the surgeon.

### Quality control of operative technique

To prevent learning curve bias, only surgeons who have performed at least 50 minimally invasive intrathoracic anastomoses and >50 cervical anastomoses can participate in this trial. To ensure the quality in each participating centre, a surgical procedure is recorded upon trial participation to evaluate the quality control of the operation. This recording was evaluated by an expert group of 10 other dedicated minimally invasive esophageal surgeons. During this meeting, this group of experts also discussed the results of MIE with CEA and MIE with IEA over the last 2 years per participating centre. Hospitals and their surgeons can only participate in the study if the experts judged both the surgical procedure and the results of the last 2 years as satisfactory. This expert opinion-based approach was used because there is currently no consensus on how surgical proficiency should be determined. Furthermore, a video will be recorded of each included patient. To ensure operative quality during the trial, the principal investigator (PI) or one of the lead investigators will evaluate the video of every fifth randomized patient. To prevent institution-related bias, only hospitals with a high volume (>30 esophagectomies per year) are invited to participate, and randomization will be stratified per hospital.

### Data collection and data management

At inclusion, a participant number will be generated, and this number will be used for further identification in the database. The participant number key is accessible by the PI and coordinating investigator. Clinical data will be collected by the study coordinator or research nurse and will be recorded in a good clinical practice (GCP)-compliant digital case record form (CRF) and database (Castor [10]). All non-electronic items containing data are kept in locked cabinets at the data coordinating centres. The data can be accessed by the research nurse, research physician and PI. Participating centres can request information from the database, but will only have access to their own centre’s data. After the study has been completed, requests to access the dataset can be submitted to the PI. The completed CRFs will also be checked with the source data regarding the primary outcome parameter and important secondary outcome parameters.

### Follow-up

For each participant, the study will start at randomization and the subject will be followed until 24 months after surgery. The primary outcome parameter will be evaluated 3 months after surgery. During the remaining 21 months of the follow-up, data on readmission, functional results, quality of life and cost-effectiveness will be generated. Study visits are scheduled to take place 2 weeks before surgery, 6 weeks after surgery and after 3, 6, 12 and 24 months.

### Strategies to improve adherence to recruitment and intervention protocols

Surgeons and local research nurses will receive email messages 1–2 days before an eligible patient visits their outpatient clinic. Inclusion rate feedback will be provided every 3 months in an electronic newsletter. Completeness of CRF data and the adherence to study protocol will be checked on a weekly basis by the coordinating investigator or research nurse, in addition to the monitoring procedure. A yearly investigators meeting will be held.

### Safety and monitoring

An independent data safety monitoring committee (DSMC) will evaluate the progress of the trial and will examine safety variables. The DSMC consists of a surgeon, a randomized controlled trial specialist and a statistician. Individualized patient description charts including safety parameters will be presented to the DSMC including one table comprising these endpoints in blinded groups for every 30 patients. The main safety parameters are all serious adverse events (SAEs) and include mortality, multiple organ failure, anastomotic leakage, pulmonary complication rate, cardiovascular complication rate, reinterventions and reoperation.

After the investigators have presented the data, the members of the DSMC will discuss these results in the absence of the investigators and will then advise them. Possible options will include continuing the trial, performing an interim analysis, adjusting the trial’s design and discontinuing the trial. Discontinuation will be advised if the DSMC concludes that the results would convince a broad range of clinicians that one trial arm is inferior or if safety is compromised in one arm. If the DSMC advises to adjust the trial’s design, to perform an interim analysis or to discontinue the trial, the responsible medical ethical committee will also be notified. SAEs will be reported to the Central Committee on Research involving Human Subjects using an online module [12].

The ICAN trial will be monitored according to the Dutch federation of universities guidelines. Since both interventions that are being investigated are considered to be standard care in the Netherlands, this is a low-risk study. The conduction of the trial will be monitored in two visits per participating site, and the monitor will check trial processes in 5 % of the included patients.

### Sample size calculation

The incidence of anastomotic leakage requiring reintervention or reoperation (type II or III [6]) after CEA is estimated to be 25 % [2, 13], and this is estimated to be 10 % after IEA [2, 3]. This estimation also corresponds to data from our own database in which data on morbidity after esophageal resection is collected from three of the participating hospitals, and this difference is considered to be clinically relevant. Based on a superiority trial design and taking an alpha of 0.05 and a power of 80 %, 100 patients per treatment arm should be included.

### Statistical analysis

Analyses will be carried out according to the intention-to-treat principle. For dichotomous data, frequencies will be presented, and continuous data will be presented as mean and standard deviation or median and range. The main outcome parameter in the two groups will be compared by calculating both the risk difference and relative risks with their 95 % confidence intervals. An additional per-protocol analysis will be done to evaluate the effectiveness of the intervention with regard to the actual treatment received. Differences in quality of life (as scored by the validated EuroQol 5D, EORTC-QLQ C30 and EORTC-QLQ OG25 questionnaires) will be analysed using a Mann-Whitney test.

Subgroup analysis will be performed on the type and configuration of anastomosis (i.e. side-to-end, side-to-side or end-to-end). Further subgroup analysis will only be carried out in case of significant interaction effects. Potential modification of the effect of each intervention will be evaluated with Poisson analyses.

Patients with open and closed procedures are replaced with new subjects. In the case of missing data, we will perform Poisson regression analyses with a robust covariance matrix estimator to adjust for covariates, since it has been shown that complete case analysis with covariate adjustment and multiple imputation yield similar estimates in the event of outcome data that are missing at random [14]. In addition, we will perform a sensitivity analysis in which a complete case analysis is compared to the multiple imputation analysis, in order to investigate whether multiple imputation would lead to different results.

## Discussion

The use of MIE is increasing worldwide, because it has been shown to reduce postoperative morbidity [15, 16]. A recent worldwide questionnaire study with 478 responders showed that approximately 55 % of surgeons prefer IEA after MIE and that there is a strong trend towards an increased use of IEA instead of CEA for reconstruction of the gastrointestinal tract [17]. However, there is currently no high-quality evidence available that favours IEA over CEA after MIE in terms of anastomotic leakage, other postoperative morbidity, functional results, quality of life or costs. In addition, IEA after MIE is a technically demanding procedure and can be accompanied by considerable postoperative morbidity, probably because of a long learning curve [18, 19]. Without evidence for a benefit of IEA over CEA and in the presence of learning curve associated morbidity, the use of an intrathoracic anastomosis as the standard reconstruction after MIE is questioned.

In theory, both anastomotic locations have possible benefits. Historically, the cervical anastomosis was introduced in order to minimize the disastrous effects of intrathoracic anastomotic leakage [20]. Cervical anastomotic leakage can sometimes be managed by bedside opening [21], although severe intrathoracic complications of cervical leaks have been described [22]. Intrathoracic anastomotic leak used to be associated with a high postoperative mortality of 60 % [21]. However, in the modern era, endoscopic, radiologic and surgical possibilities for treatment of intrathoracic anastomotic leakage have become available, and it now seems a manageable complication [23]. Benefits of the IEA are a lower incidence of anastomotic leakage [2] and possibly a lower incidence of benign strictures requiring dilatation [24–26]. A better healing of the IEA might be explained by resecting the relatively ischemic tip of the gastric tube, providing better vascularized tissue for anastomosis [21, 27]. If IEA proves to be a safe surgical technique, it might be preferred over CEA.

This study is designed to answer the question of whether an IEA or CEA is preferred after MIE. For this trial to succeed, it is essential that all participating centres are surgical experts in the creation of both IEA and CEA in order to avoid the influence of a learning curve, which is the rationale for strict centre invitation and quality control procedures. The ICAN trial will provide important data on anastomotic leakage, other postoperative morbidity, mortality, quality of life, functional results and cost-effectiveness up to 2 years after MIE. The results will aid in clinical decision for the location of the anastomosis and can provide useful information that can be incorporated into future guidelines.

### Trial status

The trial has been recruiting since May 2016.

## Abbreviations

CEA: Cervical esophagogastric anastomosis
CRF: Case record form
DSMC: Data safety monitoring committee
GCP: Good clinical practice
ICU: Intensive care unit
IEA: Intrathoracic esophagogastric anastomosis
MIE: Minimally invasive esophagectomy
